# Concurrent intracranial hemorrhage and spontaneous tumor Lysis syndrome as the initial presentation of KMT2A::AFF1-rearranged adult ALL: a case report

**DOI:** 10.3389/fonc.2026.1716717

**Published:** 2026-04-21

**Authors:** Yuyi Lai, Ligang Zhao, Min Wei, Rongguo Wei

**Affiliations:** 1 Department of Clinical Laboratory, The First People’s Hospital of Nanning, Nanning, China; 2 Department of Clinical Laboratory, The Fifth Affiliated Hospital of Guangxi Medical University, Nanning, China

**Keywords:** acute lymphoblastic leukemia, hyperleukocytosis, intracranial hemorrhage, KMT2A::AFF1 fusion, tumor lysis syndrome

## Abstract

**Background:**

*KMT2A::AFF1*-rearranged ALL is a high-risk subtype with an aggressive course. However, the simultaneous occurrence of catastrophic intracranial hemorrhage (ICH) and spontaneous tumor lysis syndrome (TLS) as the initial presentation is rare in adult patients and remains poorly characterized in the literature.

**Case Presentation:**

A previously healthy 19-year-old Chinese male was admitted with sudden-onset drowsiness and impaired consciousness. Initial computed tomography revealed multiple intracranial hemorrhages with cerebral swelling. Laboratory findings were notable for extreme hyperleukocytosis (554.66 × 10^9^/L) with 85% blasts (some with cup-like morphology), severe thrombocytopenia, and biochemical evidence of TLS. Despite emergency neurosurgical intervention and intensive supportive care, his clinical course was marked by the rapid progression of TLS and acute kidney injury, culminating in fatal cardiac arrest within 24 hours of admission. Postmortem flow cytometry and genetic analysis confirmed the diagnosis of pro-B ALL positive for the *KMT2A::AFF1* fusion gene.

**Conclusion:**

This case illustrates an exceptionally aggressive manifestation of adult *KMT2A*-rearranged ALL, characterized by the concurrent development of ICH and spontaneous TLS prior to the initiation of any leukemia-specific therapy. It underscores the critical importance of promptly recognizing this hyperacute syndrome in young patients presenting with unexplained neurological symptoms and profound hematologic abnormalities. Early multidisciplinary management and awareness of high-risk genetic profiles are essential, although the prognosis in such scenarios remains exceedingly poor.

## Introduction

Acute lymphoblastic leukemia (ALL) is a hematologic malignancy with a propensity for severe complications at presentation. Among its subtypes, the *KMT2A::AFF1* fusion gene defines a distinct high-risk entity in adults, notoriously associated with aggressive disease and poor outcomes. Although intracranial hemorrhage (ICH) and spontaneous tumor lysis syndrome (TLS) are both critical oncologic emergencies, their concurrent appearance as the initial manifestation of KMT2A-rearranged ALL is rare and typically fatal. We detail the case of a 19-year-old male who succumbed to rapidly progressive ICH and TLS within 24 hours of admission, prior to any anti-leukemic therapy, and highlight the challenges posed by this hyperacute presentation.

## Case presentation

**Figure 1:**
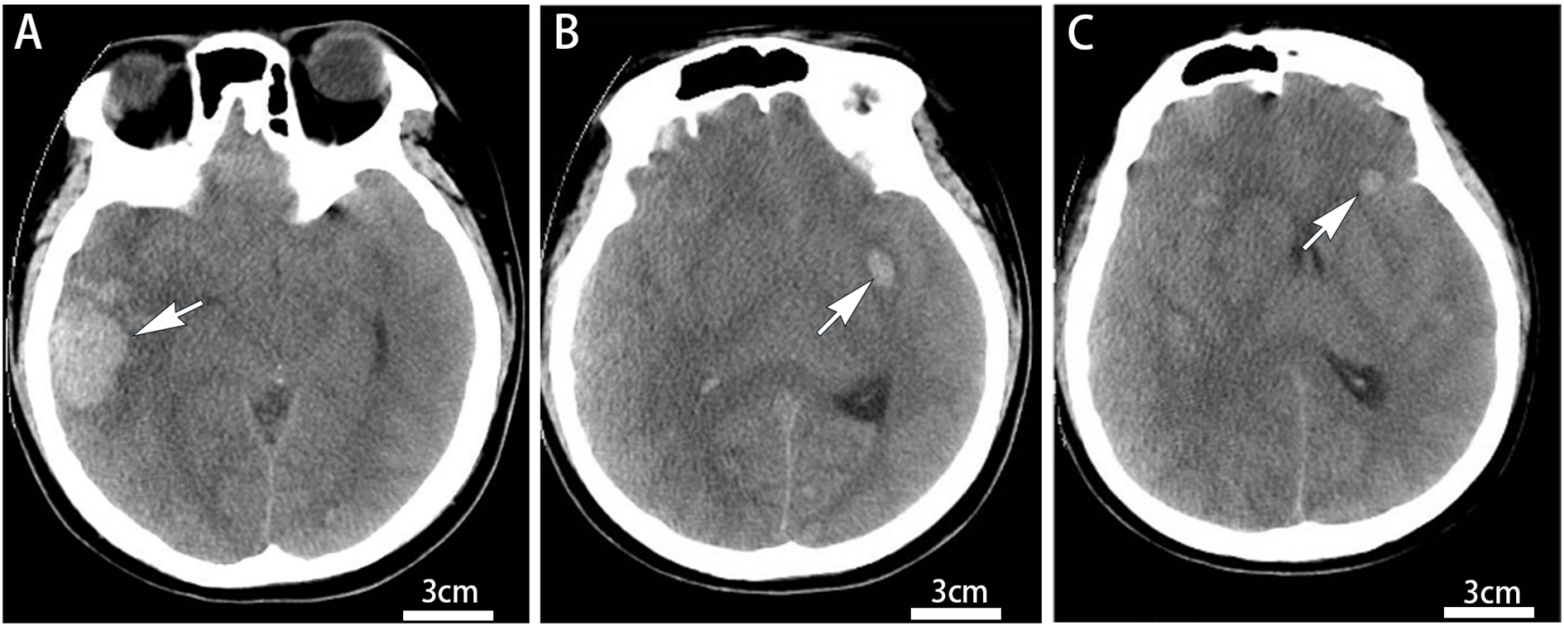
CT shows acute intracranial hemorrhages. Axial non-contrast images **(A–C)**: **(A)** right temporal lobe, **(B)** left basal ganglia, **(C)** left frontal lobe. Scale bar = 3 cm; window width 100 HU, level 35 HU. Hemorrhage volumes (ABC/2 method): **(A)** 17.6 ml, **(B)** 1.1 ml, **(C)** 0.3 ml. White arrows indicate hemorrhages.

**Figure 2:**
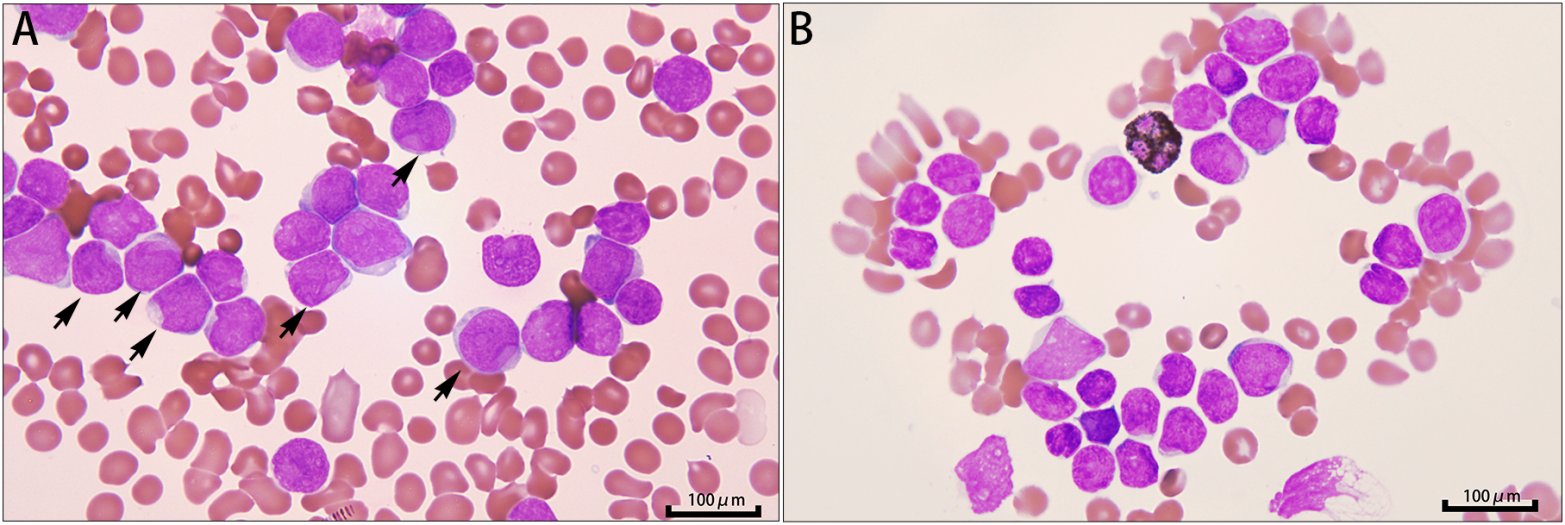
Peripheral blood smear. **(A)** Wright-Giemsa staining (1000×) identifies 85% blasts, several with cup-like morphology (black arrows). **(B)** Myeloperoxidase (MPO) stain is negative in the blasts.

A previously healthy 19-year-old Chinese male was transferred to the hospital via ambulance due to dizziness and impaired consciousness. On admission, the patient was somnolent, non-responsive to questioning, and uncooperative with physical examination. Both pupils were equal and round (3 mm in diameter) with sluggish light reflexes. Computed tomography (CT) scans revealed multiple hemorrhages in the pons and bilateral cerebral hemispheres (**Figure 1**), accompanied by cerebral edema. Additional findings included small nodules in the right middle and lower lung lobes, multiple enlarged lymph nodes in the mediastinum, bilateral axillae, and abdomen, as well as splenomegaly. Laboratory tests demonstrated a white blood cell count of 554.66×10^9^/L, with blasts accounting for 85% (some with cup-like morphology) (**Figure 2**), hemoglobin 73 g/L, platelet count 14×10^9^/L, prothrombin (PT) 13.6s, international normalized ratio (INR) 1.25, activated partial thromboplastin time (APTT) 28.2s, fibrinogen (FIB) 3.22g/L; before death, PT had prolonged to 17.2s with INR 1.58,uric acid 1291 μmol/L, and lactate dehydrogenase 1300 U/L. The patient’s clinical course was complicated by the dynamic progression of tumor lysis syndrome and renal injury (**Table 1**).

**Table 1:** Dynamic changes of TLS and renal injury.

Time point	Potassium (mmol/L)	Creatinine (μmol/L)	Uric acid (μmol/L)	Urea nitrogen (mmol/L)	INR
Admission	3.83	109	1291	7.90	1.25
2 Hours Post-op	5.94↑↑	129	1408	10.40↑	
9 Hours Post-op	6.65↑↑	237↑↑	2577↑↑	18.60↑↑	1.58↑

Symbols: ↑ indicates 1.5- to 2-fold increase from baseline; ↑↑ indicates 2- to 3-fold increase; ↑↑↑ indicates >3-fold increase.

Following admission, the patient was diagnosed with intracerebral hemorrhage, cerebral herniation, splenomegaly, acute leukemia, and hyperuricemia. The subsequent clinical course was hyperacute, with rapid progression to death. Management was initiated at 14:12 with naloxone(administered empirically for coma of unknown etiology) and hemocoagulase for hemostasis. An emergency craniotomy for hematoma evacuation and decompressive craniectomy was performed at 17:39, accompanied by intra- and post-operative transfusions of red blood cells, platelets, and fresh frozen plasma. The following day at 06:15, the patient experienced a sudden heart rate drop, followed by cardiac arrest and pulselessness. Despite immediate and sustained cardiopulmonary resuscitation, the patient failed to respond and was declared deceased at 07:46. No family history of hematologic malignancies or other cancers was reported.

**Figure 3:**
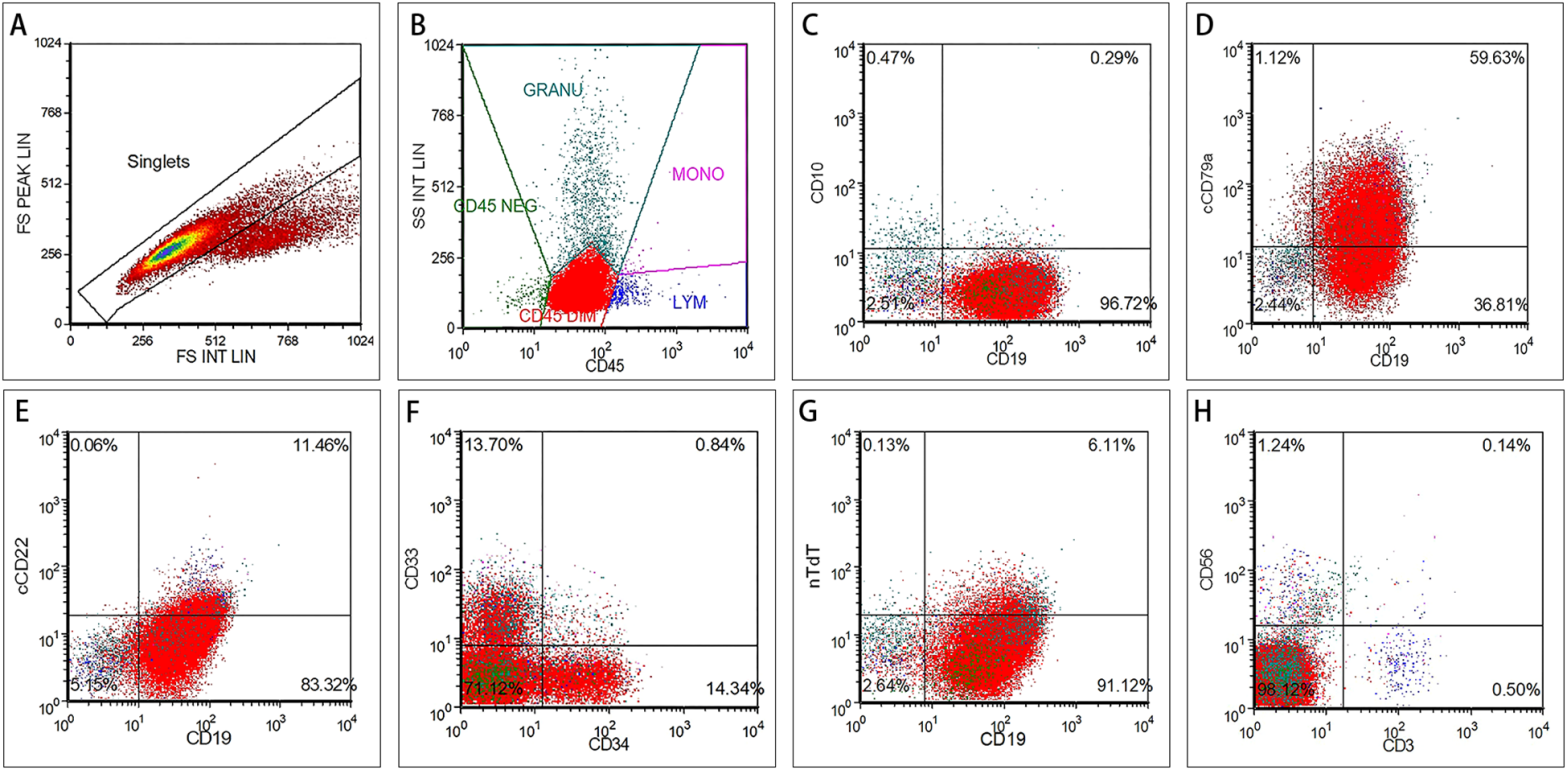
Immunophenotyping of peripheral blood blasts at diagnosis. Cells were gated on singlets **(A)** and then on the CD45^dim^/SSC^low^ blast population (**(B)**, red gate), which accounted for 96.6% of leukocytes. The gated blasts expressed CD19, were negative for CD10 **(C)**, and showed partial positivity for cytoplasmic CD79a **(D)** and cytoplasmic CD22 **(E)**. A subset of blasts aberrantly expressed CD33 on CD34^+^ cells **(F)**. nTdT was partially positive **(G)**, and T/NK markers (CD3/CD56) were negative **(H)**. Viability was 98% (7-AAD, not shown). Numbers indicate percentages.

Postmortem analysis, including peripheral blood Myeloperoxidase (MPO) staining (which was negative), flow cytometric immunophenotyping, and genetic testing, was performed to determine the cause of death. Flow cytometry revealed that 96.6% of nucleated cells were primitive/immature B lymphocytes, consistent with a diagnosis of pro-B acute lymphoblastic leukemia/lymphoma (**Figure 3**). Genetic testing confirmed the presence of the *KMT2A::AFF1* fusion gene. These findings confirmed the underlying diagnosis of KMT2A::AFF1 adult ALL with TLS and ICH.

## Discussion

This report details a 19-year-old male with *KMT2A::AFF1-*rearranged B-ALL who presented with concurrent intracranial hemorrhage (ICH) and spontaneous tumor lysis syndrome (TLS). The course was rapidly fatal, with death within 24 hours of admission.

The case is distinctive for its “dual-critical” presentation: severe thrombocytopenia and extreme hyperleukocytosis (WBC 554.66 × 10^9^/L) led to extensive brainstem ICH and rapid cerebral herniation, while the immense tumor burden triggered spontaneous TLS prior to any treatment. A temporal association was observed between neurosurgical intervention/transfusion and the rapid rise in potassium and creatinine, although causality cannot be inferred. This was followed by severe hyperkalemia and acute kidney injury within hours, culminating in cardiac arrest.

ICH typically follows vessel rupture into the brain parenchyma, caused by vasculopathy, acute blood pressure surges, or infection (1). In this patient, profound thrombocytopenia (14 × 10^9^/L) was the predominant bleeding risk factor. Hyperleukocytosis likely contributed through two mechanisms: first, less deformable leukemic blasts increased blood viscosity, causing vascular occlusion, ischemia, and endothelial injury (2); second, blast-endothelial interactions probably amplified vascular damage (2, 3). Whether the *KMT2A::AFF1* fusion independently increases hemorrhagic risk in ALL, as *KMT2A* rearrangements do through coagulopathy in AML (4), remains unknown.

The rapid progression of spontaneous TLS was further characterized in this case. By Cairo-Bishop criteria (5, 6), the patient met laboratory TLS at admission, evidenced by hyperuricemia and elevated LDH within 24 hours, this evolved into clinical TLS, marked by sharply rising creatinine and potassium levels. As Howard et al. (7) emphasized, clinical TLS (defined by acute kidney injury, cardiac arrhythmia, or death) carries a high mortality risk, consistent with this patient’s fatal outcome. The driving factor was the immense tumor load, as a white blood cell count exceeding 100 × 10^9/L is a well-established high-risk factor for TLS (5–7). Notably, the electrolyte and renal deterioration coincided temporally with platelet and red blood cell transfusions and neurosurgical intervention; however, causality cannot be established, and other contributors (e.g., perioperative ischemia, metabolic fluctuations) cannot be excluded.

The *KMT2A::AFF1* fusion gene is a pivotal genetic abnormality in adult ALL, consistently associated with aggressive disease features and poor prognosis (8–11). The aggressive clinical phenotype observed here aligns precisely with this distinct molecular profile. This fusion gene results from the chromosomal translocation t(4;11)(q21;q23), fusing the *KMT2A* gene on 11q23 with the *AFF1* gene on 4q21 (10, 12, 13). While this genetic lesion is common in infant ALL, it is found in approximately 10% of adult B-ALL cases. *KMT2A-r* ALL is characterized by hyperleukocytosis, a relatively high incidence of central nervous system (CNS) involvement, an aggressive clinical course, and a propensity for early relapse, which is a key determinant of its unfavorable outcome (10, 13). The presence of “cup-like” blasts on the peripheral blood smear, a rare but characteristic morphological marker, is also frequently associated with *KMT2A* rearrangements (14). Thus, the clinical features observed in this case—extreme hyperleukocytosis, CNS hemorrhage, and rapid clinical deterioration—are consistent with this high-risk molecular profile. This underscores the critical importance of prompt cytogenetic and molecular testing in acutely ill leukemia patients to identify such high-risk subsets.

Managing ICH, spontaneous TLS, and *KMT2A::AFF1* B-ALL concurrently presents substantial challenges. Guidelines recommend intensive blood pressure control (SBP <140 mmHg within 1 h) and timely surgery for ICH (1, 15); aggressive hydration, urate-lowering therapy (e.g., allopurinol or rasburicase based on risk), and electrolyte management for TLS (5); in this subtype, hyperleukocytosis (WBC >100×10^9^/L) may warrant prompt cytoreductive leukapheresis to rapidly reduce tumor burden. Corticosteroids, though part of standard ALL therapy, could not be used given the hyperacute presentation with concurrent immediate ICH and TLS. For targeted therapy, the first-in-class menin inhibitor revumenib was approved by the Food and Drug Administration (FDA) in 2024 for relapsed/refractory *KMT2A*-rearranged acute leukemia, with an overall response rate of 53% and a complete remission rate of 30% (16); however, data on its CNS penetration are currently unavailable, and its role in the acute setting is yet to be defined. In our patient, none of these interventions could be applied: hydration risked worsening cerebral edema; emergency craniectomy precluded leukapheresis; death occurred within 24 hours. Furthermore, diagnostic evaluation was limited: lumbar puncture was not performed, and even if it had been, concurrent ICH would have made it impossible to distinguish CNS infiltration from peripheral blood contamination. This case illustrates the therapeutic challenge when ICH and TLS occur together: treatments for one may exacerbate the other.

### Limitations

This is a single retrospective observation; postmortem diagnosis meant that antemortem molecular confirmation was unavailable to guide real-time decisions. Serial coagulation parameters were limited to two time points (on admission and before death), and continuous electrocardiographic recordings suitable for retrospective analysis were not available. Cytokine measurements were not performed. The lack of such data limits definitive attribution of cardiac arrest to hyperkalemia alone; other factors (brain hemorrhage, leukocytasis, transfusion-related metabolic changes) may have contributed. Temporal associations do not imply causation. The rarity of this presentation could not be quantified due to the absence of a systematic literature review.

## Conclusion

This fatal case illustrates that adult *KMT2A::AFF1*-rearranged B-ALL may present with simultaneous intracranial hemorrhage and spontaneous tumor lysis syndrome, creating a challenging clinical scenario. This underscores the imperative for emergency physicians to maintain a high index of suspicion for this hyperacute presentation in young patients with concurrent neurological and hematologic abnormalities. Immediate peripheral blood smear and hematology consultation are critical, and prophylactic measures against hyperleukocytosis and TLS should be initiated without delay. While this report is limited by its nature as a single case, it provides valuable insights into the aggressive nature of this leukemia subtype and supports early molecular diagnosis in such high-risk presentations.

## Data Availability

The raw data supporting the conclusions of this article will be made available by the authors, without undue reservation.

## References

[1] Seiffge DJ Fandler-Höfler S Du Y Goeldlin MB Jolink WMT Klijn CJM . Intracerebral hemorrhage — mechanisms, diagnosis and prospects for treatment and prevention. Nat Rev Neurol. (2024) 20:708–23. doi: 10.1038/s41582-024-01035-w, PMID: 39548285

[2] Hölig K Moog R . Leukocyte depletion by therapeutic leukocytapheresis in patients with leukemia. Transfus Med Hemotherapy. (2012) 39:241–5. doi: 10.1159/000341805. PMID: 22969693 PMC3434324

[3] Stucki A Rivier AS Gikic M Monai N Schapira M Spertini O . Endothelial cell activation by myeloblasts: molecular mechanisms of leukostasis and leukemic cell dissemination. Blood. (2001) 97:2121–2129. doi: 10.1182/blood.V97.7.2121, PMID: 11264180

[4] Nguyen D Kantarjian HM Short NJ Qiao W Ning J Cuglievan B . Early mortality in acute myeloid leukemia with KMT2A rearrangement is associated with high risk of bleeding and disseminated intravascular coagulation. Cancer. (2023) 129:1856–65. doi: 10.1002/cncr.34728. PMID: 36892949 PMC12013980

[5] Coiffier B Altman A Pui CH Younes A Cairo MS . Guidelines for the management of pediatric and adult tumor lysis syndrome: an evidence-based review. J Clin Oncol. (2008) 26:2767–78. doi: 10.1200/JCO.2007.15.0177. PMID: 18509186

[6] Rios-Olais FA Gil-Lopez F Mora-Cañas A Demichelis-Gómez R . Tumor lysis syndrome is associated with worse outcomes in adult patients with acute lymphoblastic leukemia. Acta Hematol. (2024) 147:391–401. doi: 10.1159/000534453. PMID: 37963436

[7] Howard SC Jones DP Pui CH . The tumor lysis syndrome. N Engl J Med. (2011) 364:1844–54. doi: 10.1056/NEJMra0904569. PMID: 21561350 PMC3437249

[8] Smith AL Denny N Chahrour C Sharp K Arachi M Dopico-Fernandez A . Enhancer heterogeneity in acute lymphoblastic leukemia drives differential gene expression in patients. Blood. (2025) 146:2073–2087. doi: 10.1182/blood.2024028019. PMID: 40729681 PMC7618588

[9] Richard-Carpentier G Kantarjian HM Tang G Yin CC Khoury JD Issa GC . Outcomes of acute lymphoblastic leukemia with KMT2A (MLL) rearrangement: the MD Anderson experience. Blood Adv. (2021) 5:5415–9. doi: 10.1182/bloodadvances.2021004580. PMID: 34525185 PMC9153023

[10] Piciocchi A Messina M Elia L Vitale A Soddu S Testi AM . Prognostic impact of t(4;11)(q21;q23)/KMT2A-AFF1-positivity in 926 BCR-ABL1-negative B-lineage acute lymphoblastic leukemia patients treated in GIMEMA clinical trials since 1996. Blood. (2019) 134:1469. doi: 10.1182/blood-2019-121977. PMID: 34048072

[11] Yin L Wan L Zhang Y Hua S Shao X . Recent Developments and Evolving Therapeutic Strategies in KMT2A-Rearranged Acute Leukemia. Cancer Medicine (2024) 3:e70326. doi: 10.1002/cam4.70326, PMID: 39428967 PMC11491690

[12] Peterson JF Smoley SA Luoma IM Pitel BA Rice CS Benevides Demasi JC . Characterization of a cryptic KMT2A/AFF1 gene fusion by mate-pair sequencing (MPseq) in a young adult with newly diagnosed B-lymphoblastic leukemia. Journal of Hematopathology. (2019). 12:99–104. doi: 10.1007/s12308-019-00355-x, PMID: 30311153

[13] Górecki M Kozioł I Kopystecka A Budzyńska J Zawitkowska J Lejman M . Updates in KMT2A gene rearrangement in pediatric acute lymphoblastic leukemia. Biomedicines. (2023) 11:821. doi: 10.3390/biomedicines11030821. PMID: 36979800 PMC10045821

[14] Sayyada A Bhat A Sundaram UD Chadha R . Cup-Like Blasts in B-Lymphoblastic Leukemia with KMT2A Rearrangement: A Rare Morphological Presentation. Indian Journal of Hematology and Blood Transfusion. (2025) 42:290–293. doi: 10.1007/s12288-025-02048-1, PMID: 41522563 PMC12789309

[15] Wang S Wang J Elmadhoun O Liang Y Zhao W . Advances in intracerebral hemorrhage management: A comprehensive review. Brain Circ. (2026) 12:13–21. doi: 10.4103/bc.bc_177_24. PMID: 41815808 PMC12974928

[16] Al Dali S Al-Mashdali AF Kalfah A Mohamed SF . Menin inhibitors in KMT2A-rearranged and NPM1-mutated acute leukemia: a scoping review of safety and efficacy. Crit Rev Oncol Hematol. (2025) 213:104783. doi: 10.1016/j.critrevonc.2025.104783. PMID: 40441466

